# Supplementary material for: Understanding reticence to occupy free, novel-design homes: A qualitative study in Mtwara, Southeast Tanzania
Source: PLOS Glob Public Health. 2023 Nov 22;3(11):e0002307. doi: 10.1371/journal.pgph.0002307 (PMC10664911; doi:10.1371/journal.pgph.0002307)
Supplement: Attachment — Submitted filename: Response to Reviewers Comments.docx [file pgph.0002307.s002.docx]

**Response to Reviewers Comments**

Please use the space provided to explain your answers to the questions above. You may also include additional comments for the author, including concerns about dual publication, research ethics, or publication ethics. (Please upload your review as an attachment if it exceeds 20,000 characters)

**Reviewer #1**:

The findings of the study are very intriguing. This study highlights the continued prevalence of superstitions in underprivileged and poor communities. The ‘results’ section is well written. However, there are a few points that I would like to raise

1. A few studies should be mentioned in the third paragraph of the ‘background’ section that previously investigated the existing superstitious concepts like ‘freemansons’ and supernatural forces in the poor and uneducated communities in Tanzania or Africa.

References have been added. We however return to a more detailed discussion of the accusations and link with broader literature in the discussion section.

2. The interview guide of the in-depth interview should be discussed lucidly.

*More text has been added to elaborate on the topics included in the guides for the specific respondent groups and how these were used during the data collection.*

3. The detailed process of the focus group discussions (FGDs) is not mentioned. The authors should mention how the FGDs were conducted, what questions were discussed with the respondents.

*Additional text has been added to outline the topics covered in the focus groups and how the guides were used in a flexible and iterative manner during the data collection.*

4. The findings correspond to the objectives of the study. However, it appears that interpretations are written repeatedly in the paragraphs no 2-6 of the ‘discussion’ section. I would like to see it rewritten in a crisp manner not repeating certain things.

*These paragraphs have been heavily edited and re-structured to make them more concise and improve reading.*

5. Nowhere in the discussion section I see the mention of role of low or little education behind the reluctance and fear of the residents in Mtwara to occupy the free, novel-design houses. It should be interpreted.

*We did not include a discussion of education level of the participants because we were unable to collect information on the educational status of those making the accusations or acting on them, particularly because the accusations were usually described indirectly. We have made general statements about the lack of research experience of the communities. Although the overall education level of the communities is likely not high, it should also be noted that similar sorts of rumours have been recorded among more educated groups. We have added a sentence to this effect in the last paragraph of the discussion.*

**Reviewer #2:**

This manuscript presents and interprets qualitative findings from a housing intervention study, where the study experienced challenges in acceptance and use of the newly constructed houses by community members.

I found the paper to be well written, and appreciate the authors' openness to share the challenges experienced during this trial, so that their experiences may inform and guide other researchers.

I have only a couple of minor suggestions:

1. In line 155 an eligibility survey is mentioned, and in line 138 it is stated that the target families were the poorest members of the community, even though that was not the intention. Could you unpack this a little, perhaps stating in line 155 the specific eligibility criteria for a household to be entered into a lottery? Were the vast majority of households in each village considered eligible, or only a small subset? This may also help to contextualise the later statements about envy of those who received the new homes

An extra line has been added here – yes, most but not all households were eligible based on the type of housing. The additional requirement for two children under the age of 13 years was not met by many of these households.

2. Line 168. Suggest being consistent with the pilot study location name - here it is Tanga, previously it was Magoda.

We have removed the reference to Tanga pilot to avoid confusion and now refer to the pilot alone. (The previous pilot study was conducted in Tanga, whereas the trial is underway in Magoda)

3. Line 298. This sentence requires rewording

*This sentence has been adjusted.*
